# Integrated Multiscale Appearance Features and Motion Information Prediction Network for Anomaly Detection

**DOI:** 10.1155/2021/6789956

**Published:** 2021-10-20

**Authors:** Ting Liu, Chengqing Zhang, Liming Wang

**Affiliations:** ^1^State Key Lab for Electronic Testing Technology, North University of China, Taiyuan 030051, China; ^2^College of Mechatronics Engineering, North University of China, Taiyuan 030051, China

## Abstract

The rise of video-prediction algorithms has largely promoted the development of anomaly detection in video surveillance for smart cities and public security. However, most current methods relied on single-scale information to extract appearance (spatial) features and lacked motion (temporal) continuity between video frames. This can cause a loss of partial spatiotemporal information that has great potential to predict future frames, affecting the accuracy of abnormality detection. Thus, we propose a novel prediction network to improve the performance of anomaly detection. Due to the objects of various scales in each video, we use different receptive fields to extract detailed appearance features by the hybrid dilated convolution (HDC) module. Meanwhile, the deeper bidirectional convolutional long short-term memory (DB-ConvLSTM) module can remember the motion information between consecutive frames. Furthermore, we use RGB difference loss to replace optical flow loss as temporal constraint, which greatly reduces the time for optical flow extraction. Compared with the state-of-the-art methods in the anomaly-detection task, experiments prove that our method can more accurately detect abnormalities in various video surveillance scenes.

## 1. Introduction

Due to the corona virus disease of 2019 (COVID-19) outbreak, many countries have been accelerating the construction of smart cities and public-safety systems [1] to efficiently manage surrounding circumstances. As part of these systems, traditional video surveillance systems rely on manual monitoring to find abnormalities in massive video data. This operation increases working time, labor costs, and misjudgments. Therefore, automatic detection of anomalous behaviors [2] has attracted increasing researcher attention because of its potential application values. An intelligent video surveillance system aims to provide a supervisor with precise anomaly cues to deal with abnormal events as soon as possible. However, it is a highly challenging task in the computer vision field, because anomaly detection suffers two core issues. First, only normal samples are readily available during the training phase because of the rare occurrence of abnormal events in most cases. Second, anomalous events are various and complicated, and the definition of “abnormality” heavily depends on the context; hence, there is no standard definition. It is difficult to mark abnormal events and detect these behaviors using supervised technology.

To solve the aforementioned problems, most state-of-the-art approaches adopt unsupervised techniques and then use regular events as training samples to train the model. When the test sample deviates significantly from the learned model, it is detected as an anomaly. To date, the large variety of anomaly-detection methods can be roughly divided into two types: (1) hand-crafted feature approaches and (2) deep-learning approaches. In hand-crafted feature methods, the core idea is mainly to adopt hand-crafted features to represent video sequences. These features include trajectory features [3] and low-level features (e.g., histograms of oriented gradients [4], histograms of optical flow [5], and 3D gradients [6]). They are heavily dependent on the feature-extraction process and expert knowledge, which directly limit the accurate representation of complex feature patterns and affect the accuracy of anomaly detection. Deep-learning approaches commonly use reconstruction error-based methods. These methods follow the rule that normal events produce a small reconstruction error, whereas abnormal events generate a large error. They evaluate the anomaly based on the consistency between the generated and the input frames. Specifically, Hasan et al. [7] presented an approach based on the auto-encoder that reconstructs regularities with low error but incurs higher reconstruction error for irregularities. However, because a convolution operation is only used for feature extraction, this structure cannot model temporal information in a long video sequence. Consequently, Chong and Tay [8] and Luo et al. [9] added convolutional long short-term memory (ConvLSTM) layers to the auto-encoder for performing the memory of temporal information. Li Chang [10] presented a multivariate Gaussian fully convolution adversarial auto-encoder (MGFC-AAE) to model gradient and optical flow patches for anomaly detection. George et al. [11] proposed a nonuniform spatiotemporal region resembling parallelepipeds to extract the histogram of optical flow orientation and magnitude features. These approaches simultaneously modelled spatial and temporal features from the input data, making them more suitable for video analysis. Nevertheless, it is challenging to obtain a large reconstruction error for anomalies owing to the powerful learning capacity of a deep neural network. Moreover, because of the self-reconstructed generated frames, the methods identify anomalies regardless of context information. Therefore, high missed and false detection phenomena occur while executing these methods.

Considering the shortcomings of reconstruction approaches, some researchers have begun to use video-prediction algorithms, namely, future-frame prediction based on a sequence of previous video frames, to detect abnormal behaviors. These methods agree with the idea that normal events are predictable, whereas abnormal events are unpredictable. By only training regular events to obtain a prediction model, anomalies in videos refer to events that rarely or should not occur in a particular scenario. For example, Munawar et al. [12] created a deep prediction network to detect the abnormal operation behaviors of industrial robots. Villegas et al. [13] combined LSTM and analogy-based encoder-decoder networks to tackle long-term video-prediction tasks from a hierarchical perspective. Additionally, Zhao et al. [14] proposed a spatiotemporal auto-encoder involving the three-dimensional (3D) convolution for video anomaly detection. Nevertheless, these methods based on an auto-encoder structure use only single-scale information from the previous layer in the decoding process, leading to the detailed information loss for the different-size objects in the videos. Thus, Liu et al. [15] proposed a method to predict future frames on the basis of U-Net, which can effectively retain the multiscale structural characteristics of the input frames by the skip connection. However, the conventional U-Net cannot adequately consider the motion continuity between video frames.

Motivated by the aforementioned anomaly detection task, it is necessary to sufficiently consider multiscale spatial features and temporal continuity for recognizing abnormal behaviors. Recently, lots of works have achieved great detection performance by using multiscale features of images; for example, Gao et al. [16] adopted multiscale single-stage object detector for pose detection in the classroom scene. Oh et al. [17] proposed multiscale convolutional recurrent neural network for inspecting and classifying bearing fault defects. The literatures [18, 19] used multiview receptive field network for foreground detection. Owing to the camera position and angle, objects multiscale features extraction can effectively improve the performance of target detection. In this paper, we propose a novel spatiotemporal prediction network, i.e., STP-net, which fuses the multiscale appearance features and motion information extraction module. The main idea is to utilize the network to model the video content and internal dynamic changes by training the ordinary events accurately. If the test-video prediction frame is significantly different from the actual frame, an abnormality is detected. First, we use the HDC module [20] to extract multiscale spatial features and learn the objects scale variations. Then, we adopt the DB-ConvLSTM [21] module to memorize the temporal information and obtain the complex motions features between consecutive frames. Finally, we perform the predicted future frame from the spatial and temporal dimensions. At the same time, the literatures [22, 23] showed that RGB difference is a valid substitute for optical flow [24] as a new type of temporal loss. This operation could achieve a similar effect but significantly reduce the computational cost to extract optical flow information.

Specifically, the main contributions of our work are as follows:

1. Starting from the second downsampling of U-Net, the HDC module acts on the previous convolution layer of each downsampling layer to increase the convolution kernel receptive field, making it easy to retain more data detailed information and improve the representational capacity of the model.
2. At the end of the encoding process of U-Net, the DB-ConvLSTM strategy can take full advantage of the relationship between consecutive frames to extract detailed temporal information, which can strengthen the temporal continuity between the video frames and effectively improves the accuracy of the prediction results.
3. Experimental results on several public benchmark datasets indicate the superior ability of our method compared with the state-of-the-art approaches in the abnormality detection task.

The remainder of this paper is organized as follows.  Section 2 provides the overall framework of the proposed method.  Section 3 elucidates and discusses the experimental validation through a series of primary public datasets. Finally, Section 4 summarizes the general conclusions and discusses future research directions.

## 2. Proposed Method

**Figure 1:**
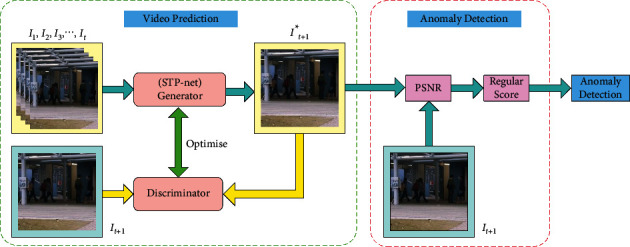
Overall framework of the proposed method.

As shown in Figure 1, the overall framework of our method can be divided into two parts: video prediction and anomaly detection. The first part aims to train a generator network to predict future frames. To generate a high-quality prediction frame, we use the generative adversarial network (GAN) [25] and several loss functions to optimize our network model. We treat the STP-net as generator network (G) and then adopt frames (*I*_1_, *I*_2_, *I*_3_,…, *I*_*t*_) before the current frame *I*_*t*+1_ as the input tensor, and the predicted frame *I*_*t*+1_^*∗*^ as the output tensor. For the discriminative network (D), we choose PatchGAN [26] to strengthen the recognizing ability between the actual and generated frames. Finally, we use the total objective optimization function to minimize the distance between the predicted frame and the target frame, making *I*_*t*+1_^*∗*^ closer to *I*_*t*+1_. In the second part, we employ the pretrained model to judge the extent of abnormality by calculating each frame's regular score. Next, we will illustrate the different components of the proposed framework in detail.

### 2.1. Video Prediction

**Figure 2:**
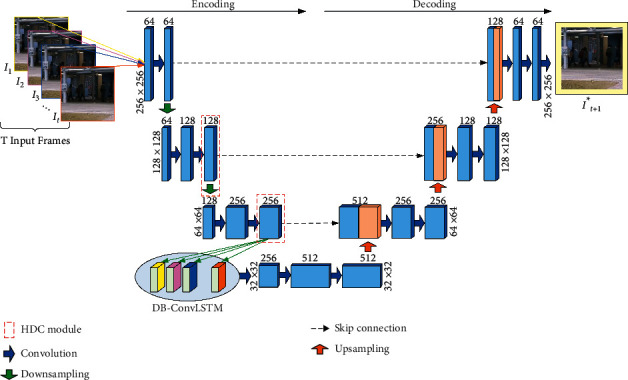
The structure of STP-net. The resolutions of feature maps are equal in the same layer.

On the basis of U-Net structure, the details of STP-net are presented in Figure 2. We add HDC module to extract multiscale spatial features of the training samples and then insert DB-ConvLSTM to handle temporal information between the continuous *T* frames in a nonlinear manner. The network comprises an encoding path and a decoding path. The input and output size of the network are both 256 × 256 × 3. The kernel sizes of all convolution and deconvolution are set to 3 × 3 and the maxpool layers are set to 2 × 2.

#### 2.1.1. Multiscale Features Extracted Strategy

The objects forms and sizes are different owing to the camera position and angle. Inspired by the HDC applied in the semantic segmentation field, it is essential to consider multiscale feature information. Meanwhile, the multiple downsampling operations of the U-Net will lead to the severe loss of spatial detailed information. In order to improve the network's learning ability, we should not only consider extracting multiscale spatial information, but also consider compensating for the loss due to the downsampling operation; thus, starting from the second downsampling, the HDC module acts on the previous convolution layer of each downsampling layer to retain more image detail information. The reason why HDC is not used before the first downsampling layer is that several convolution operations before first downsampling will not cause a lot of loss to image information.

**Figure 3:**
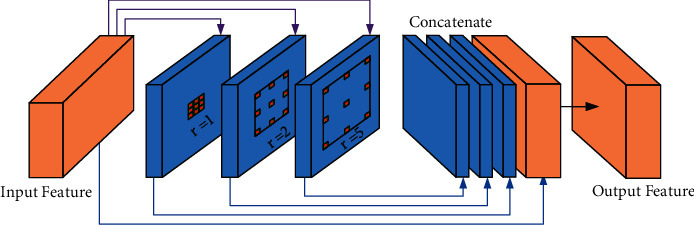
The structure of HDC module. The sizes of input feature maps are 128 × 128 × 128 and 64 × 64 × 256. The dilation rates are set to 1, 2, and 5, respectively.

The structure of HDC module is shown in Figure 3. The input feature maps are fed into three different branches. These branches are used to acquire the different size of receptive field and automatically extract multiscale features through a set of dilated convolutions with different dilation rates. It is also worth mentioning that a small dilation rate is fit for extracting features of small objects, while a large dilation rate is fit for obtaining features of large objects. Finally, the features from each branch are concatenated with the input feature maps for enhancing contextual information and multiscale spatial features representation.

#### 2.1.2. Temporal Information Extracted Strategy

The current anomaly detection methods usually adopt three-dimensional (3D) convolution or ConvLSTM [27] to extract the temporal correlation of the input data. The 3D convolution requires more computational time to process a large number of model parameters. Therefore, lots of researchers choose ConvLSTM structure for time modelling. However, the ConvLSTM can only remember the sequence data in the forward direction. According to study [21, 28], it is evident that considering both forward and backward feature information is important and complementary for predicting future frames. Thus, we use DB-ConvLSTM module to capture more comprehensive spatiotemporal characteristics.

**Figure 4:**
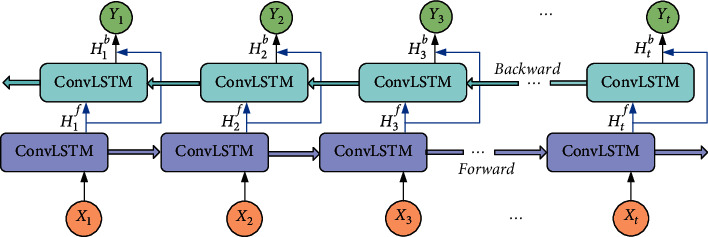
The structure of DB-ConvLSTM module.

The input mode of our network is different from existing methods that conventionally stack *T* consecutive frames together into a network. In these methods, all the *T* frames are connected to each channel in the first output feature map, which results in the collapse of temporal information [29]; thus, we input *T* frames into the encoder network one by one to generate corresponding feature maps. As shown in Figure 4, the DB-ConvLSTM structure includes a shallow forward layer and a deeper backward layer. Specifically, {*H*_*t*_^*f*^} denotes the corresponding outputs of forward sequential feature maps from the ConvLSTM units in the forward layer. The deeper backward layer receives the forward sequential outputs {*H*_*t*_^*f*^} to generate {*H*_*t*_^*b*^} corresponding outputs of backward sequential feature maps. Then, we use equation (1) to process the forward and the backward features maps to obtain the final output sequence {*Y*_*t*_}. Finally, the information can exchange between the forward and backward directional ConvLSTM units to capture more powerful and complementary spatiotemporal features. As shown in Figure 4, we feed the last output *Y*_*t*_ containing both spatial features and relevant temporal features into the decoding process.

(1) $$\begin{array}{c} Y_{t}=\tanh\left(W_{y}^{H^{f}}*H_{t}^{f}+W_{y}^{H^{b}}*H_{t}^{b}+b\right). \end{array}$$

#### 2.1.3. Loss Function

We used spatial and temporal constraints to optimize the model and minimize the difference between the predicted frame and its ground truth. The intensity constraint can guarantee the similarity of all pixels in the RGB space, and the gradient constraint can sharpen the generated images. Therefore, we chose intensity and gradient constraints as spatial constraint to promote the predicted frames *I* ^*∗*^ to be consistent with the corresponding ground truth *I*. Moreover, the temporal loss defined as the RGB difference between the prediction frame and the ground truth guarantees the correctness of motion prediction for anomaly detection. The intensity loss, gradient loss, and temporal loss are defined as equations (2)–(4), respectively.

(2) $$\begin{array}{c} L_{\mathrm{int}}\left(I^{*},I\right)={\left\|I^{*}-I\right\|}_{2}^{2}, \end{array}$$

(3) $$\begin{array}{c} L_{gd}\left(I^{*},I\right)=\sum_{i,j}{\left\|\left|I_{i,j}^{*}-I_{i-1,j}^{*}\right|-\left|I_{i,j}-I_{i-1,j}\right|\right\|}_{1}+{\left\|\left|I_{i,j}^{*}-I_{i,j-1}^{*}\right|-\left|I_{i,j}-I_{i,j-1}\right|\right\|}_{1}, \end{array}$$

(4) $$\begin{array}{c} L_{rgb}\left(I^{*},I\right)={\left\|\left|I_{t+1}^{*}-I_{t}\right|-\left|I_{t+1}-I_{t}\right|\right\|}_{1}. \end{array}$$

We also leveraged GAN to constrain the training process owing to its excellent image generation [30] and video-prediction [31] performance in recent years. Specifically, *G* attempts to generate future frames that are as realistic as possible, whereas *D* aims to distinguish the frames generated by G. Ideally, the goal of the GAN is to reach the Nash equilibrium. When training *D*, the procedure aims to classify *I∗* into class 0 and *I* into class 1, where 0 represents the generated frame, and 1 indicates the genuine frame. The loss function used to train *D* is imposed as equation (5). When training *G*, the goal is to let the generated frames *I∗* classified into class 1 by D. Then, the adversarial loss for *G* is defined as shown in equation (6):

(5) $$\begin{array}{c} L_{adv}^{D}\left(I^{*},I\right)=\frac{1}{2}{\left(D\left(I^{*}\right)-0\right)}^{2}+\frac{1}{2}{\left(D\left(I\right)-1\right)}^{2}, \end{array}$$

(6) $$\begin{array}{c} L_{adv}^{G}\left(I^{*}\right)=\frac{1}{2}{\left(D\left(I^{*}\right)-1\right)}^{2}. \end{array}$$

To obtain a well-trained model that has a better ability to identify abnormalities, we considered all the aforementioned constraints, such as spatial, temporal, and adversarial training loss, into our final objective function (7). During training *D*, we fixed the weights of *G* to optimize objective function (8).

(7) $$\begin{array}{c} L_{G}=\alpha_{\mathrm{int}}L_{\mathrm{int}}+\alpha_{gd}L_{gd}+\alpha_{rgb}L_{rgb}+\alpha_{adv}L_{adv}^{G}, \end{array}$$

(8) $$\begin{array}{c} L_{D}=L_{adv}^{D}, \end{array}$$

where *α*_*int*_, *α*_*gd*_, *α*_*rgb*_, and *α*_*adv*_ are coefficients for the corresponding constraints, respectively.

### 2.2. Anomaly Detection

After training the model to represent regular events in video sequences, we used the difference between the predicted frame *I*^*∗*^ and ground truth *I* for anomaly prediction. To the best of our knowledge, Peak Signal to Noise Ratio (PSNR) [32] is widely used to assess the image quality as follows:

(9) PSNRI∗,I=10  log10maxI∗21/N∑i=0NIi∗−Ii2,

where *I*^*∗*^ represents the predicted frame, *I* denotes the corresponding ground truth, max_*I*_*∗*__ represents the maximum value of the image intensities, *N* represents the total number of pixels, and *i* represents the pixel index.

In the test phase, we chose the PSNR to evaluate the predicted frame. A higher PSNR value means that the predicted frame is more similar to its ground truth and indicates that it is more likely to be a regular event and vice versa. For comparison, we normalized the PSNR of all frames in each test video to the range [0, 1], and the regular score can be calculated as

(10) $$\begin{array}{c} S\left(t\right)=\frac{\text{PSNR}\left(I_{t}^{*},I_{t}\right)-\min_{t}\text{PSNR}\left(I_{t}^{*},I_{t}\right)}{\max_{t}\text{PSNR}\left(I_{t}^{*},I_{t}\right)-\min_{t}\text{PSNR}\left(I_{t}^{*},I_{t}\right)}, \end{array}$$

where the min_*t*_PSNR and max_*t*_PSNR are the minimum and maximum values of the PSNR in every test video frame, respectively.

## 3. Experimental Results and Discussion

In this section, we validate the proposed method performance on publicly available benchmark datasets, including the Chinese University of Hong Kong (CUHK) Avenue dataset [33] and the University of California San Diego (UCSD) Pedestrian dataset [34]. We further utilize the recorded real video data to verify the robustness of our model. The proposed framework was implemented by PyTorch and supported by an NVIDIA Tesla V100.

### 3.1. Evaluation Metric

**Figure 5:**
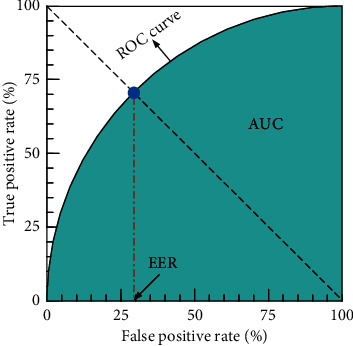
Relationship between AUC and EER.

To validate the effectiveness of the proposed method, we followed the performance evaluation of frame-level criteria. We selected the receiver operating characteristic (ROC) curve as an indicator to evaluate the anomaly detection algorithms. The ROC curve is obtained by gradually changing the threshold and calculating the true positive rate (TPR) and the false positive rate (FPR). In this study, our approach is compared with the existing anomaly-detection methods using the area under the curve (AUC) and equal error rate (EER). Higher AUC values and lower EER values indicated better anomaly detection performance. The relationship between AUC and EER is illustrated in Figure 5.

### 3.2. Dataset Description

CUHK Avenue Dataset is collected on Campus Avenue at the Chinese University of Hong Kong and includes 16 training videos (15,328 training frames) and 21 testing videos (15,324 testing frames). Each video-frame resolution is 360 × 640 pixels, and the frame rate for each video clip is 25 frames per second. Normal events are mainly behaviors of pedestrians walking on the sidewalk. The anomalies include abnormal events, such as running, loitering, and throwing objects.

UCSD Dataset contains two subsets, Ped1 and Ped2, which comprise videos collected by the University of California San Diego from public pedestrian areas taken at different viewing angles. Ped1 comprises 34 training scenes and 36 testing scenes with a frame resolution of 238 × 158 pixels. Ped2 includes 16 training scenes and 12 testing scenes with a frame resolution of 360 × 240 pixels. Ped1 and Ped2 have the same definitions of normal and abnormal events. In regular videos, some pedestrians walk on the sidewalk. However, in abnormal cases, these are bicycles, vehicles, skateboarders, and wheelchairs crossing pedestrian areas.

### 3.3. Training Details

For the training details of our model, we adopted Adam [35] to train the network for parameter optimization. We set *T* to 4, used a random clip of five sequential frames, and set the mini-batch size to 4. For greyscale datasets, we set the learning rates of the generator and discriminator to 0.0001 and 0.00001, while we set them to 0.0002 and 0.00002 for color-scale datasets. For different datasets, the coefficient factors *α*_*int*_, *α*_*gd*_, *α*_*rgb*_, and *α*_*adv*_ were slightly different.

### 3.4. Performance Analysis of the Proposed Method

**Figure 6:**
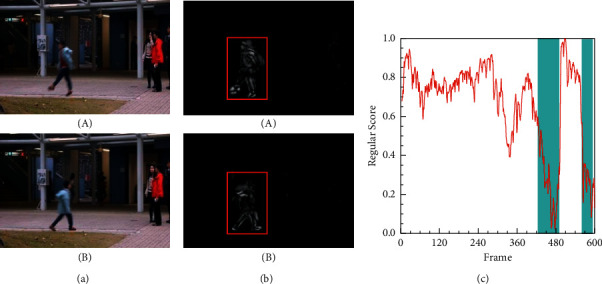
Frame-level evaluation results on Avenue 7th test video. (a) Ground truth of the 465th frame (A) and 593rd frame (B) labelled as abnormal. (b) Difference between the ground truth and the corresponding predicted frame. (c) Relationship between the test video frames and the regular score.

We analyze the corresponding experimental results of different datasets. For a better illustration, in Figure 6, specific events are chosen to display the anomaly detection results from the seventh test video on the Avenue dataset.  Figure 6(a) shows the corresponding ground truth.  Figure 6(b) presents the difference between the ground truth and the corresponding predicted frames.  Figure 6(c) displays the relationship between the test video frames and the regular score. The blue blocks represent the ground truth annotation of frames containing abnormal events, and the red line represents the regular score of every frame. As shown in Figure 6(c), higher regular scores represent the usual events. In comparison, the lower regular scores corresponding to the blue area are the abnormal events shown in Figure 6(a) (e.g., the child running from a different direction). When executing the prediction model, our method has learned prior information and then predicts what will happen next. Under the pedestrian street scene, the model gains the appearance and motion features of walking persons from the training samples. As shown in Figure 6(b), when the testing frames of a running person are fed into the model, it can only predict a person while walking, which generates a big difference (labelled with a red rectangle) between the predicted frame and the ground truth.

**Figure 7:**
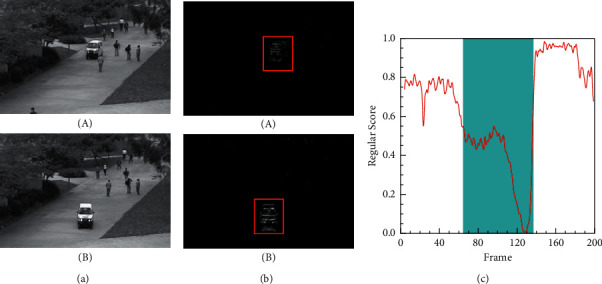
Frame-level evaluation results on Ped1 19th test video. (a) Ground truth of the 91st frame (A) and 118th frame (B) labelled as abnormal. (b) Difference between the ground truth and the corresponding predicted frame. (c) Relationship between the test video frames and the regular score.

**Figure 8:**
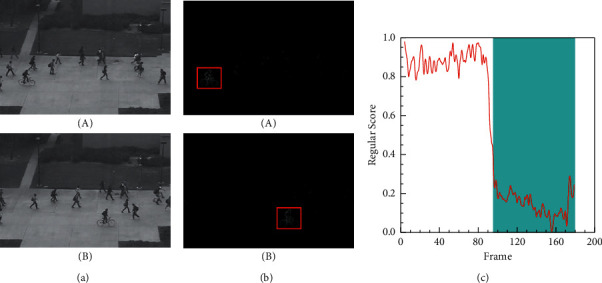
Frame-level evaluation results on Ped2 2nd test video. (a) Ground truth of the 109th frame (A) and 160th frame (B) labelled as abnormal. (b) Difference between the ground truth and the corresponding predicted frame. (c) Relationship between the test video frames and the regular score.

The size and shape of the objects may change because of the different position and angle of the camera. More specifically, Figures 7 and 8 show the detection results of anomalous events from different video angles on the UCSD Ped1 and Ped2 datasets. The illustrations of these figures are similar to Figure 6. As shown in Figures 7(a) and 7(b) and 8(a) and 8(b), objects located close to the camera appear to be larger than those far from it, although they are the same objects. Moreover, we can see that our method can easily detect abnormal events (e.g., cars and cyclists) from different situations. As shown in Figures 7(c) and 8(c), the lower regular scores are consistent with the ground truth labelled as abnormal events (e.g., the cars in the Ped1 19th test video and the cyclists in the Ped2 2nd test video). Higher regular scores indicate normal events. After analyzing the experimental data, we find that our method is robust when facing these different types of spatial features, because it uses the advantages of HDC module to pay more attention to the multiscale spatial characteristics.

**Figure 9:**
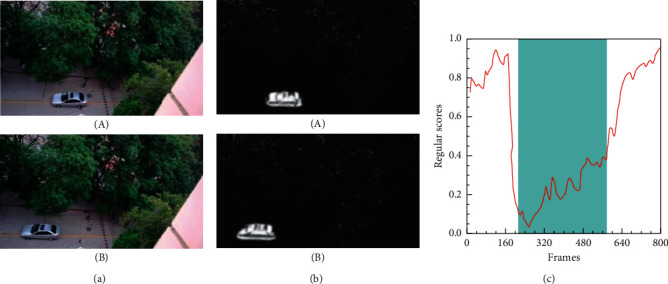
Frame-level evaluation results on the recorded real video. (a) Ground truth of the 432th frame (A) and 496th frame (B) labelled as abnormal. (b) Difference between the ground truth and the corresponding predicted frame. (c) Relationship between the recorded real video frames and the regular score.

To validate that our method is actually working on a real scenario, we recorded the street scene next to our building and verified the proposed model. The illustrations of these figures are similar to Figure 6. As shown in Figures 9(a) and 9(b), we can see that our method can easily detect abnormal events (e.g., car) from the recorded real video. As shown in Figure 9(c), higher regular scores represent normal activities. The lower regular scores are consistent with the ground truth labelled as abnormal activity.

**Figure 10:**
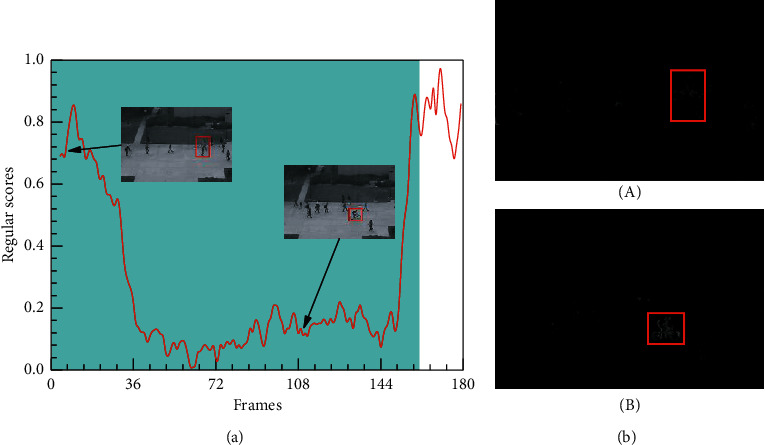
Frame-level evaluation results on Ped2 6th test video. (a) Relationship between the test video frames and the regular score. (b) Difference between the ground truth and the corresponding generated frame.

Additionally, Figure 10 shows the experimental failure case of detecting anomalies in the initial stage on the UCSD Ped2 dataset. As shown in Figure 10(a), we can see that abnormal events (e.g., occluded cyclist) cannot be detected, but the cyclist can be detected without occlusion. The higher regular scores are consistent with the ground truth labelled as abnormal events in the initial phase. As shown in Figure 10(b), the difference (occluded cyclist labelled with a red rectangle) between the ground truth and the corresponding generated frame is ambiguous, but the other one is clear. After analyzing the experimental data, it is worth mentioning that our method might not perform well, because the abnormal events could be temporally occluded by other objects in the video. The main attention of our future work is to solve the problem caused by occlusion, by exploiting visual tracking technology to tackle the miss detection in highly occlusion scenes.

### 3.5. Performance Comparison of Different Methods

**Figure 11:**
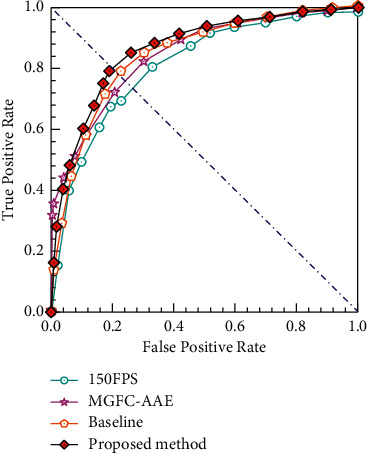
ROC curves comparison of different methods on Avenue dataset.

To intuitively display the changing trend of ROC curves of different methods in terms of the frame-level criterion, Figure 11 depicts the results of our method compared with three typical approaches, e.g., MGFC-AAE [10], Baseline [15], and 150FPS [33] on the Avenue dataset. We can observe that the ROC curve of our method is significantly higher than that of the other algorithms.  Table 1 presents a quantitative comparison of our method with other recently published approaches for AUC values. Compared with these approaches, the proposed method achieved the highest AUC value, which reached 86.4%, demonstrating good performance.

**Figure 12:**
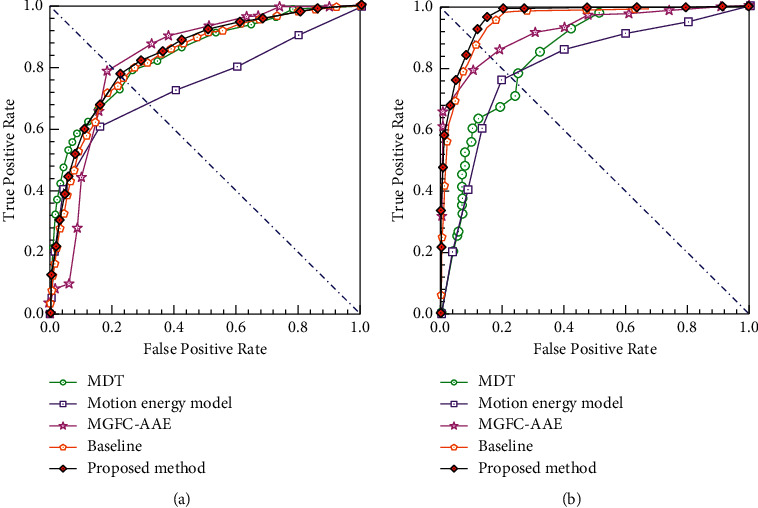
ROC curves comparison of different methods on UCSD dataset. (a) Frame-level ROC on Ped1. (b) Frame-level ROC on Ped2.

Figures 12(a) and 12(b) depict the comparison results of the ROC curves of different methods on the UCSD dataset. We chose some deep-learning algorithms [10, 15] and traditional methods [34, 38], e.g., MGFC-AAE [10], Baseline [15], mixtures of dynamic textures (MDT) [34], and motion energy model [38]. From the comparison, we can see that our method outperforms most of the existing methods. The experimental results further demonstrate the superiority of the deep-learning methods compared with the traditional methods.  Table 2 lists the detailed quantitative comparison data of the different algorithms in the aspect of the AUC metric. We set the literature [15] as the baseline during the evaluation phase because of its excellent performance for anomaly detection based on a prediction network. In detail, our method raises 1.3% and 0.9% for Ped1 and Ped2 datasets compared with Baseline [15]. In conclusion, our method is effective for detecting anomalies on the UCSD dataset.

Through the aforementioned comparison, the proposed method achieved better results in various video surveillance scenes; the AUC value obtained by our model is superior to most existing models. For a more comprehensive analysis, we also adopted EER as the evaluation metric.  Table 3 presents the detection results obtained from the proposed method as well as other methods. It can be seen from the data that our method reaches a lower EER compared with all other methods except ConvLSTM [8] (Ped1) and AnomalyNet [37] (Ped2). The experimental results demonstrate the superiority of our approach in anomaly detection task.

Moreover, we choose the more typical per sample prediction time (i.e., average running time comprises the prediction frame generation and anomaly detection) to evaluate the complexity of the proposed solution.  Table 4 shows the running time of our approach in comparison with several previous methods on UCSD Ped2 dataset. It can be seen that our method is a little bit slow than MDT [34] and Unmasking [36], but the AUC value obtained by our model is superior to these methods. Besides, our approach runs almost as fast as baseline [15]. The reason lies in that we add the HDC module and the DB-ConvLSTM module, which takes time. In general, our method can ensure running time and accuracy to be better working on a real world.

### 3.6. Ablation Studies

To verify the effectiveness of each component of the proposed method, we conducted an ablation study for different component. For comparison, three variants of the proposed method (i.e., STP-net only with HDC, with ConvLSTM, and with DB-ConvLSTM) were trained to evaluate the performance for anomaly detection.  Table 5 shows the AUC values obtained from the variants with different component on the different datasets. It can be observed that the variant with all components achieves the best results than those with fewer components, which shows the importance to take full advantage of the spatiotemporal features for anomaly detection. The HDC module can extract the more representative multiscale spatial features, and the DB-ConvLSTM module can memorize the temporal information. The experimental results indicate the effectiveness of our method, which fully considers spatiotemporal information.

In addition, we evaluated the effect of optical flow loss and RGB difference loss for our model on different datasets. As shown in Tables 6 and 7, when the RGB difference loss was employed in the network, the average runtime using batch data reduced from 0.4648 (s/batch) to 0.0036 (s/batch) and the AUC values significantly improve by 0.6% (Avenue), 0.5% (UCSD Ped1), and 0.6% (UCSD Ped2), respectively. It is obvious that the RGB difference loss replaced optical flow loss can greatly save the time of optical flow extraction and shorten the training time. In summary, our method gives a full consideration of the spatiotemporal information, thus effectively improving the accuracy of the detection results.

## 4. Conclusions and Future Work

This paper proposes an effective anomaly-detection method based on the STP-net by integrating HDC and DB-ConvLSTM module. We employ the proposed network to capture more comprehensive multiscale spatial features and temporal information of regular events. In the testing stage, the abnormalities of the test video were detected by the lower regular scores calculated by the PSNR values between the predicted frames and actual frames. Furthermore, using RGB differences as motion loss can reduce the training time. To further evaluate the proposed model, we conducted a series of experiments on several public benchmark datasets. The experimental results show that the AUC values of the CUHK Avenue, UCSD Ped1, and Ped2 datasets reached 86.4%, 84.4%, and 96.3%, respectively. Our method performs well compared with the state-of-the-art approaches in terms of detection accuracy through qualitative analysis and quantitative comparisons.

The proposed method does not limit the type of abnormality, and it can achieve the general detection of different abnormal behaviors in a specific scenario. Therefore, our method can be conveniently applied to various video surveillance scenarios. However, this approach still has some shortcomings and limitations. First, the prediction method is highly dependent on prior information; thus, the detection results are sensitive to any changes of the previous frame. Second, our method might perform poorly on fairly easy to detect the abnormalities due to the occluded abnormal events. Third, the prediction network relies on the completeness of training data, implying that the training data should contain all normal behaviors of the scenario. To develop a complete anomaly detection system, as part of the future scope, we plan to exploit visual tracking technology to solve the problem of sensitivity and occlusion. Meanwhile, we will extend existing datasets to cover as many different surveillance video scenarios as possible to address smart-city and public-security issues.

## Figures and Tables

**Table 1:** Frame-level AUC performance of different methods on Avenue dataset.

Methods	AUC (%)
150FPS [33]	80.9
Conv-AE [7]	70.2
ConvLSTM [8]	80.3
ConvLSTM-AE [9]	77.0
MGFC-AAE [10]	84.2
Unmasking [36]	80.6
AnomalyNet [37]	86.1
Baseline [15]	84.9
Proposed method	86.4

**Table 2:** Frame-level AUC performance of different methods on UCSD dataset.

Methods	AUC (%)	
	UCSD Ped1	UCSD Ped2
MDT [34]	81.8	82.9
Motion energy model [38]	75	81
Conv-AE [7]	81.0	90.0
ConvLSTM [8]	89.9	87.4
ConvLSTM-AE [9]	75.5	88.1
MGFC-AAE [10]	85	91.6
Unmasking [36]	68.4	82.2
AnomalyNet [37]	83.5	94.9
Baseline [15]	83.1	95.4
Proposed method	84.4	96.3

**Table 3:** Comparison of EER performance on different datasets.

Methods	EER (%)		
	UCSD Ped1	UCSD Ped2	Avenue
Conv-AE [7]	27.9	21.7	25.1
ConvLSTM [8]	12.5	12	20.7
MGFC-AAE [10]	20	16	22.3
AnomalyNet [37]	25.2	10.3	22
Baseline [15]	24	12	21
Proposed method	22.8	11	19.7

**Table 4:** Comparison of running time performance on UCSD Ped2 dataset.

Method	Running time (frames per second)
MDT [34]	23
Unmasking [36]	20
Baseline [15]	32
Proposed method	29

**Table 5:** Effect of different components on AUC values.

Components	AUC (%)		
	Avenue	UCSD Ped1	UCSD Ped2
HDC	85.4	83.8	95.7
ConvLSTM	85.2	83.5	95.4
DB-ConvLSTM	85.5	83.9	95.6
HDC and DB-ConvLSTM	86.4	84.4	96.3

**Table 6:** Effect of different type loss functions on runtimes.

Loss function	Running time (s/batch)		
	Avenue	UCSD Ped1	UCSD Ped2
With optical flow loss	0.4685	0.4643	0.4615
With RGB difference loss	0.0036	0.0036	0.0036

**Table 7:** Effect of different type motion loss function on AUC values.

Loss function	AUC (%)		
	Avenue	UCSD Ped1	UCSD Ped2
With optical flow loss	85.8	83.9	95.7
With RGB difference loss	86.4	84.4	96.3

## Data Availability

The original data used to support the findings of this study are included in the article.
